# Supplementary figures and images for: Coral larvae increase nitrogen assimilation to stabilize algal symbiosis and combat bleaching under increased temperature
Source: PLoS Biol. 2024 Nov 12;22(11):e3002875. doi: 10.1371/journal.pbio.3002875 (PMC11556732; doi:10.1371/journal.pbio.3002875)

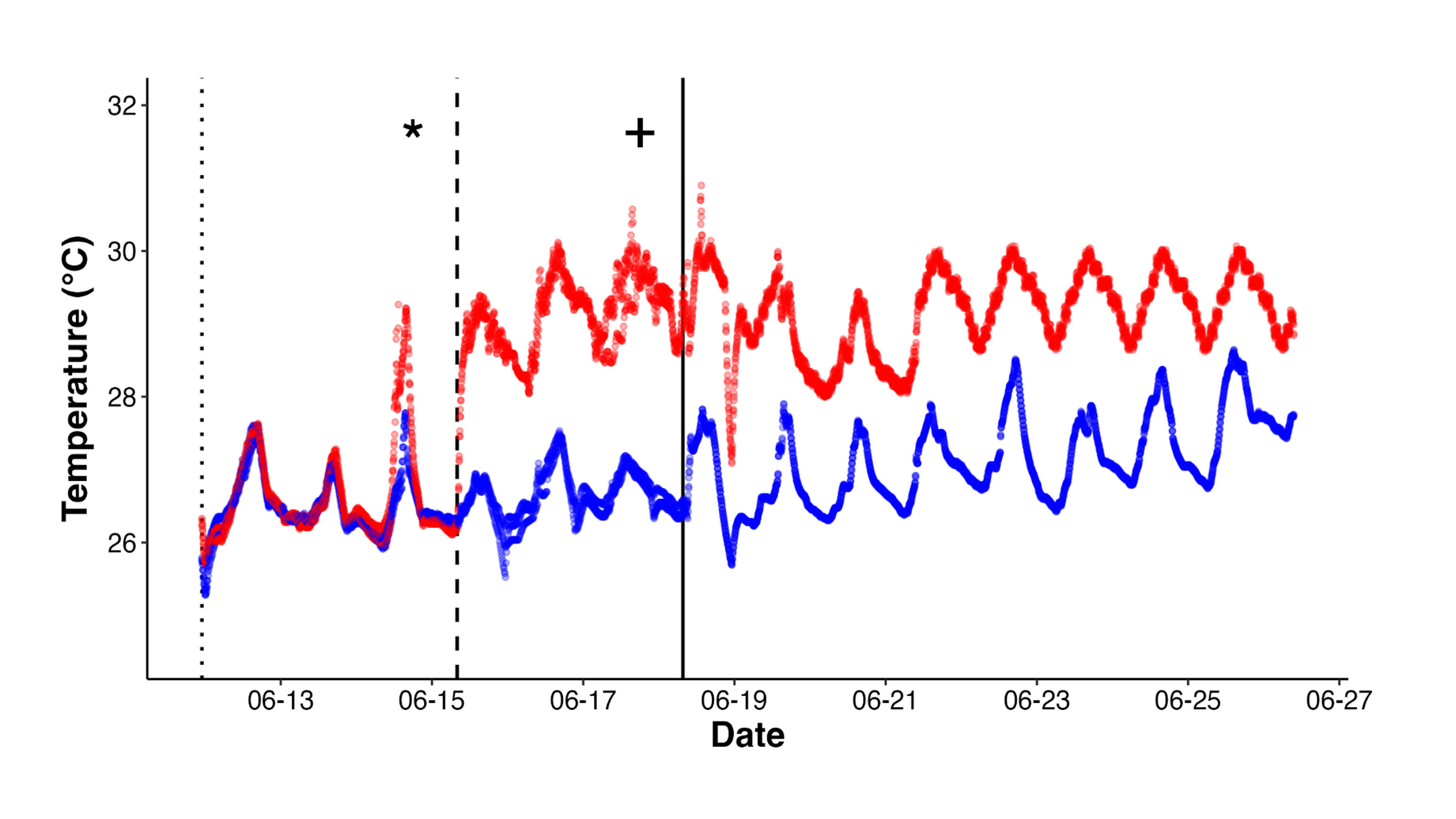

Supplement: S1 Fig — X-axis indicates date (MM-DD). Asterisks (*) indicate the start of temperature treatments (06–14) and plus (+) indicates time of larval sampling (06–18). Temperature recorded every 15 min by n = 3 loggers per temperature treatment. Each point represents individual temperature measurements recorded by each logger. The data underlying this figure can be found at 10.5281/zenodo.13835295. (TIFF) [file pbio.3002875.s001.tiff]

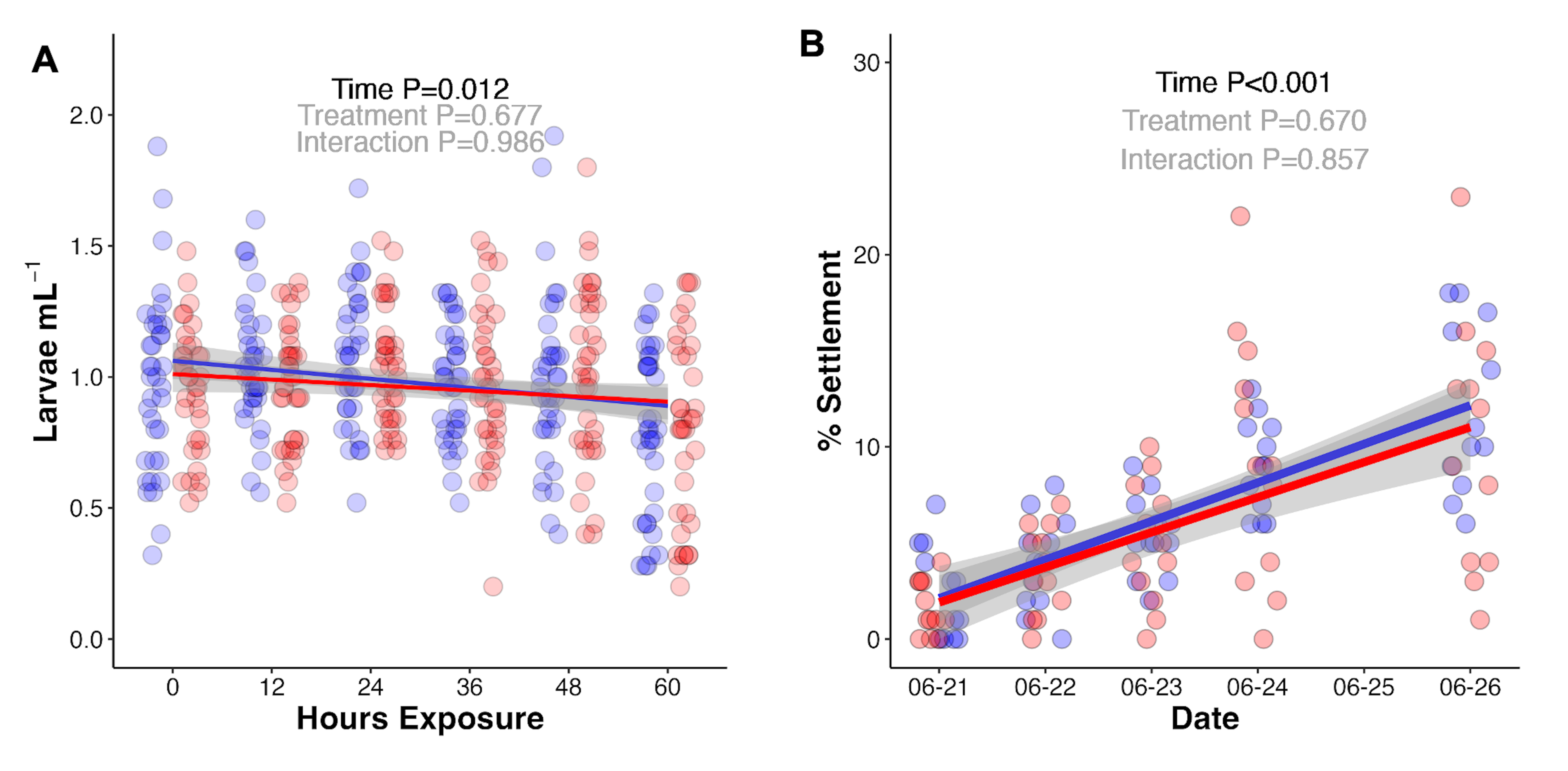

Supplement: S2 Fig — (B) Percent larval settlement. Following larval exposure, larvae were settled under temperature treatments and held at these treatments over a 5-day period. Effects of time and rearing treatment were tested using linear mixed effect models with tank as a random intercept (black text P < 0.05; gray text P > 0.05). In both plots, points represent individual replicates. Linear model fit lines shown with gray indicating 95% confidence intervals. The data underlying this figure can be found at 10.5281/zenodo.13835295. (TIFF) [file pbio.3002875.s002.tiff]

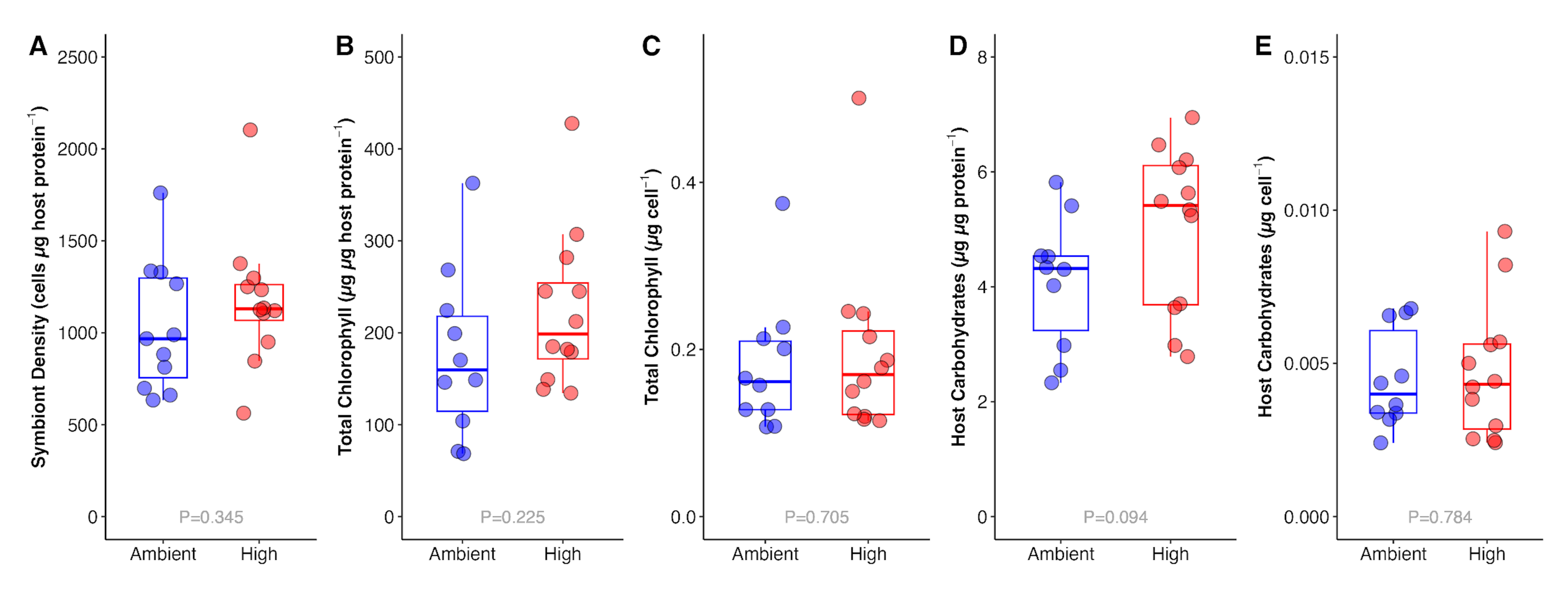

Supplement: S3 Fig — (A) Symbiont cell density, cells per μg host protein content. (B) Symbiont chlorophyll (a + c2), μg per μg host protein content. (C) Symbiont chlorophyll (a + c2), μg per symbiont cell. (D) Host carbohydrate content, μg per μg host protein content. (E) Host carbohydrate content, μg per symbiont cell. For all responses, effect of treatment was tested using Welch t tests (black text P < 0.05; gray text P > 0.05). In all plots, point represent individual replicates. The data underlying this figure can be found at 10.5281/zenodo.13835295. (TIFF) [file pbio.3002875.s003.tiff]

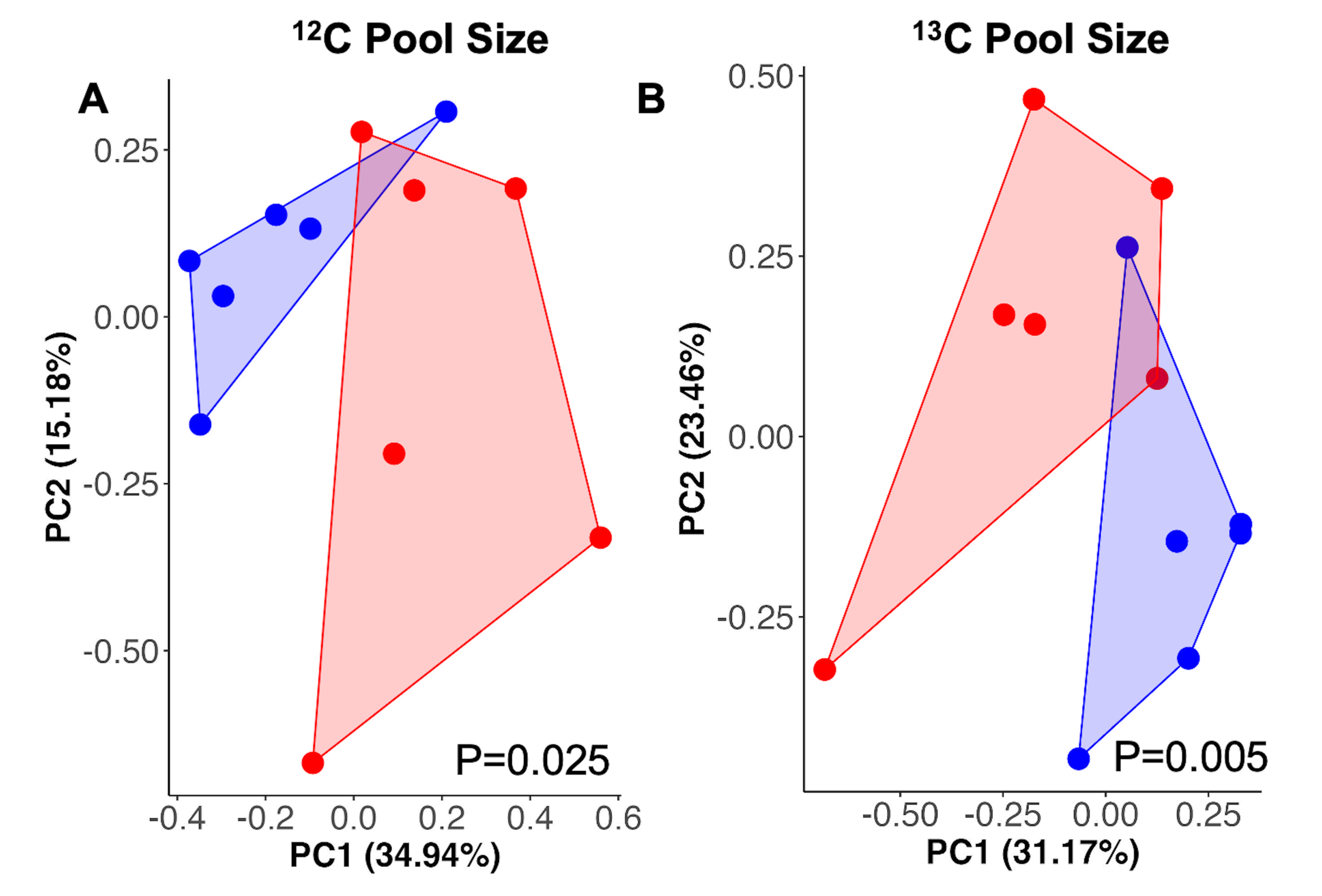

Supplement: S4 Fig — (A) Principal components analysis of metabolite pool sizes in larvae incubated with unlabeled (12C) sodium bicarbonate between temperature treatments. (B) Principal components analysis of metabolite pool sizes in larvae incubated with labeled (13C) sodium bicarbonate between temperature treatments. Blue indicates larvae exposed to ambient temperature; red indicates larvae exposed to high temperature. Axis show percent variance explained by each principal component. P-values indicate significance of temperature treatment on multivariate pool size analyzed using PERMANOVA analyses. The data underlying this figure can be found at 10.5281/zenodo.13835295. (TIFF) [file pbio.3002875.s004.tiff]

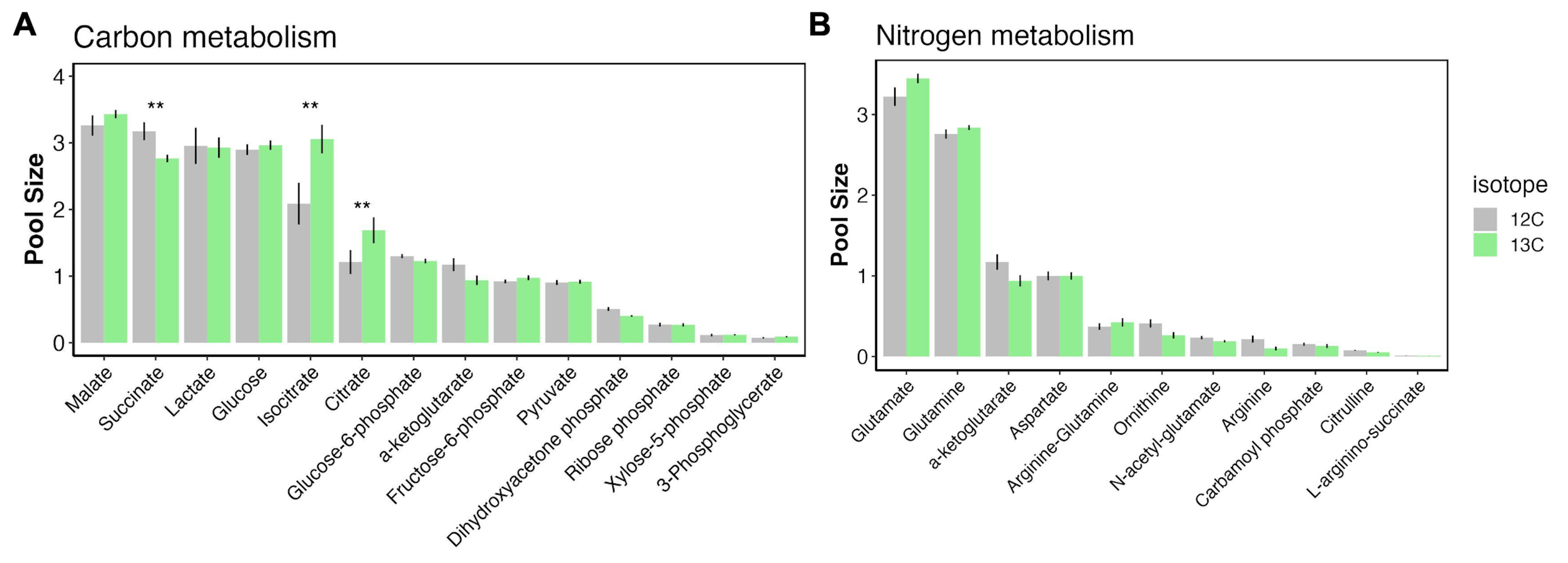

Supplement: S5 Fig — (B) Mean (± standard error of mean) pool size in metabolites of interest related to nitrogen metabolism, including ammonium assimilation and the urea cycle in 12C (gray) and 13C (green) isotope treatments. In all plots, error bars represent standard error of mean. ** indicates P < 0.01 as determined by estimated marginal means post hoc tests. No asterisks indicate P > 0.05. The data underlying this figure can be found at 10.5281/zenodo.13835295. (TIFF) [file pbio.3002875.s005.tiff]

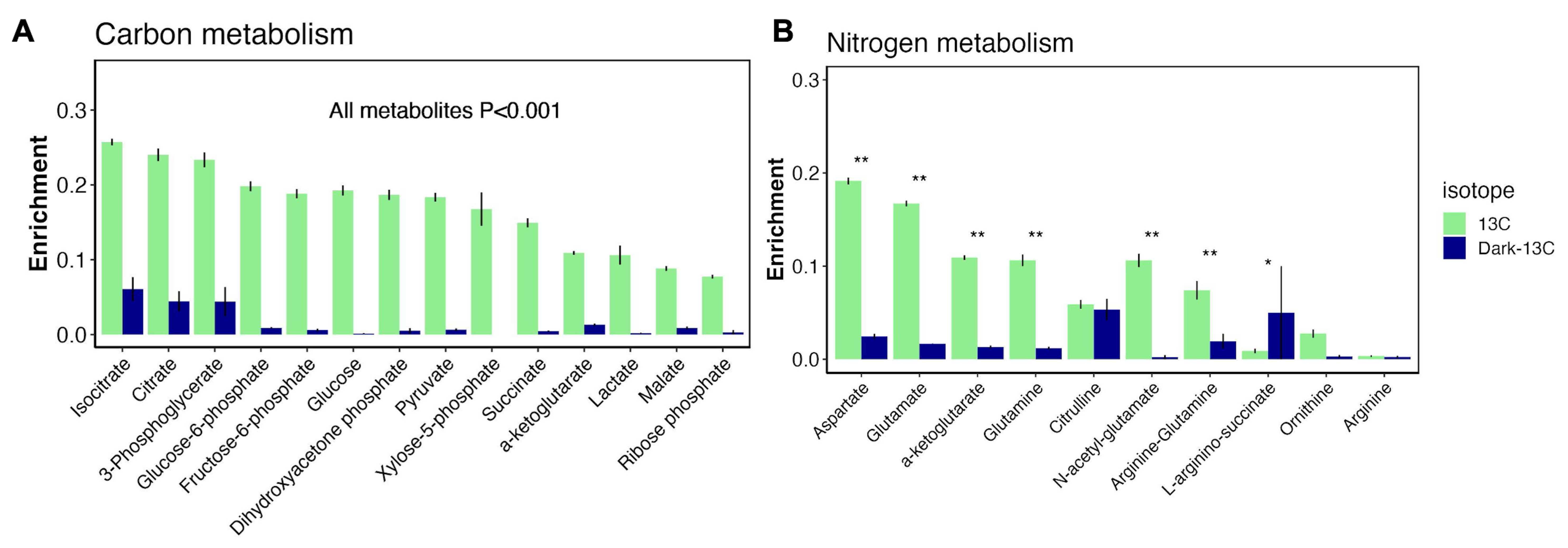

Supplement: S6 Fig — Mean (± standard error of mean) enrichment in metabolites of interest related to (A) carbon (including glycolysis, pentose phosphate pathway, and the tricarboxylic acid cycle) and (B) nitrogen metabolism (including ammonium assimilation and the urea cycle) in larvae incubated with labeled 13C sodium bicarbonate in the light (green) and the dark (blue). * Indicates P < 0.05 and ** indicates P < 0.01 as determined by estimated marginal means post hoc tests. No asterisks indicate P > 0.05. In (A), all metabolites were significant at P < 0.001. The data underlying this figure can be found at 10.5281/zenodo.13835295. (TIFF) [file pbio.3002875.s006.tiff]

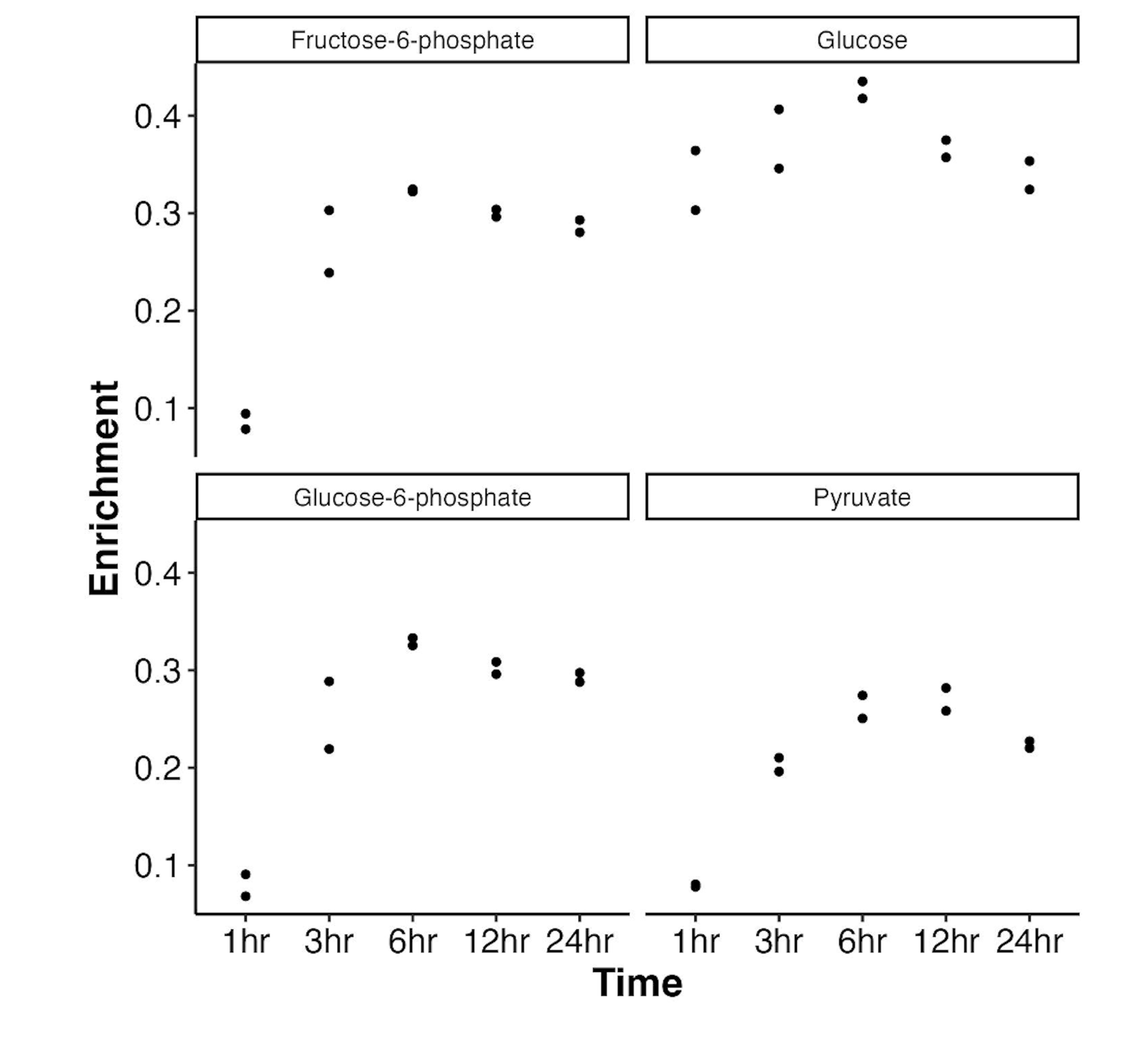

Supplement: S7 Fig — Isotopic label enrichment shown for glucose (a primary photosynthate), glycolysis intermediates (fructose-6-phosphate, glucose-6-phosphate), and a glycolysis product (pyruvate) across a 24 h time series sampling. Points represent individual replications. The data underlying this figure can be found at 10.5281/zenodo.13835295. (TIFF) [file pbio.3002875.s007.tiff]

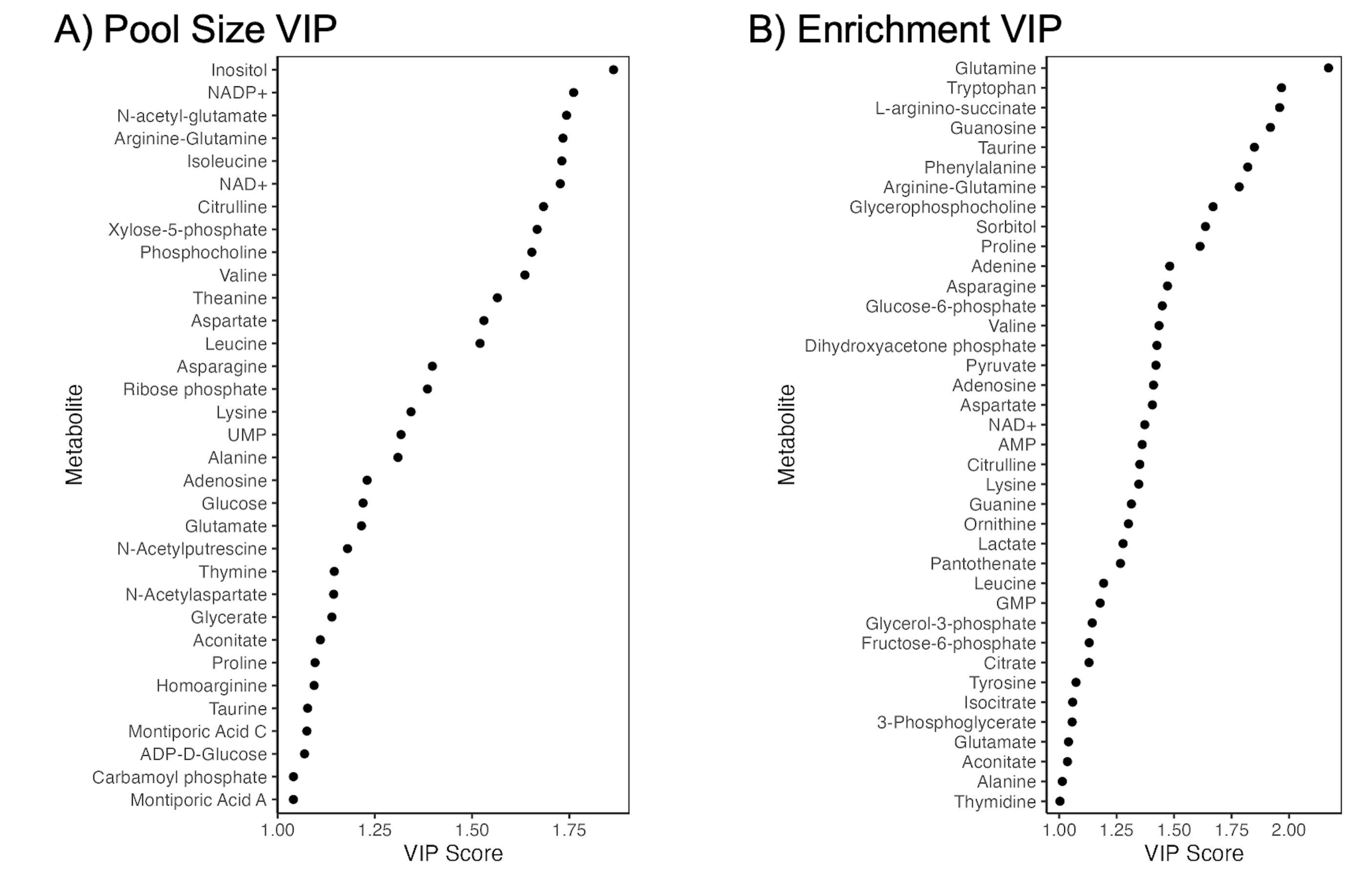

Supplement: S8 Fig — VIPs were identified through partial least squares discriminant analysis (PLS-DA). The data underlying this figure can be found at 10.5281/zenodo.13835295. (TIFF) [file pbio.3002875.s008.tiff]

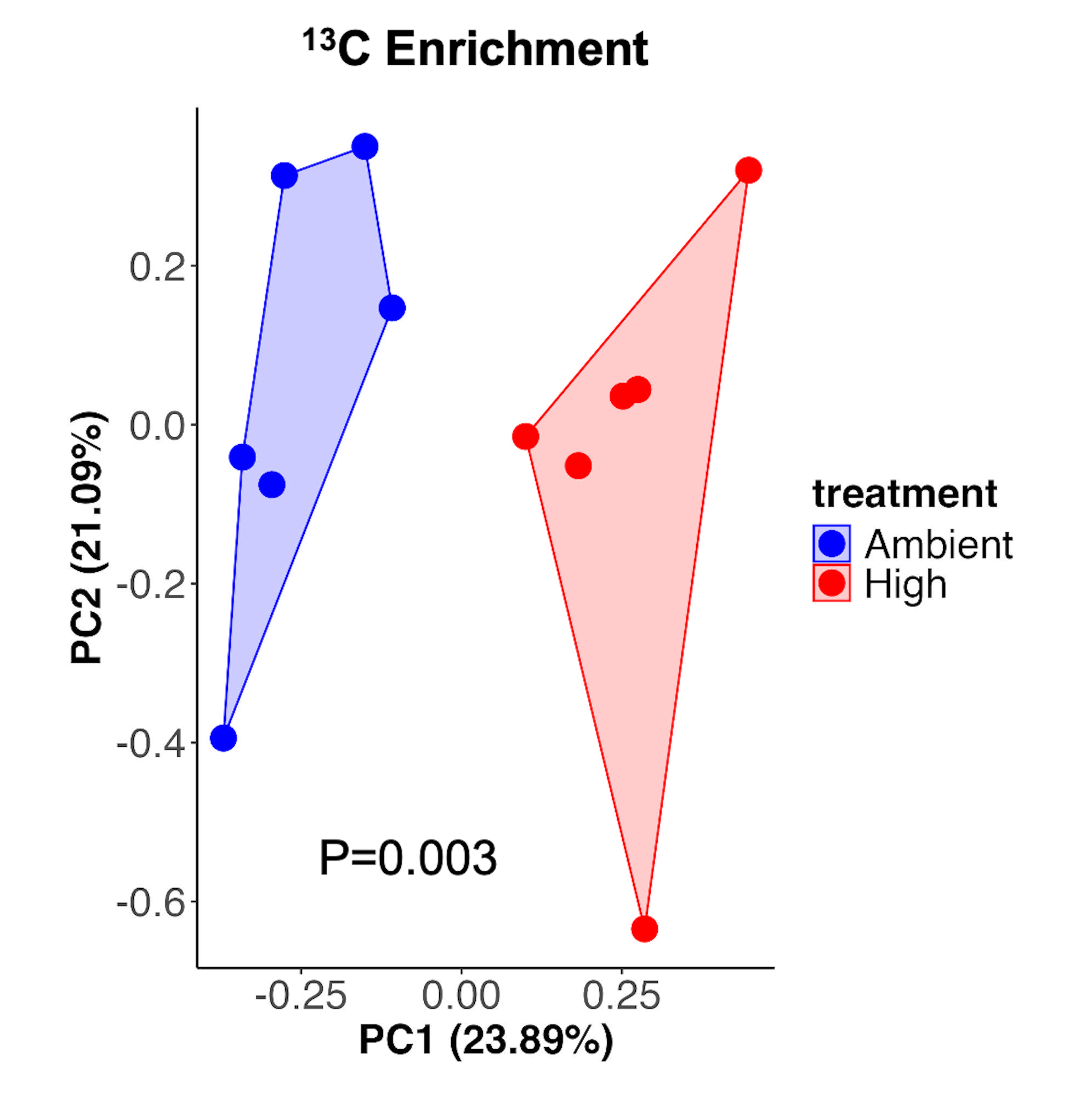

Supplement: S9 Fig — Axes show percent variance explained by each principal component. P-value indicates significance of temperature treatment on multivariate enrichment analyzed using PERMANOVA analyses. The data underlying this figure can be found at 10.5281/zenodo.13835295. (TIFF) [file pbio.3002875.s009.tiff]

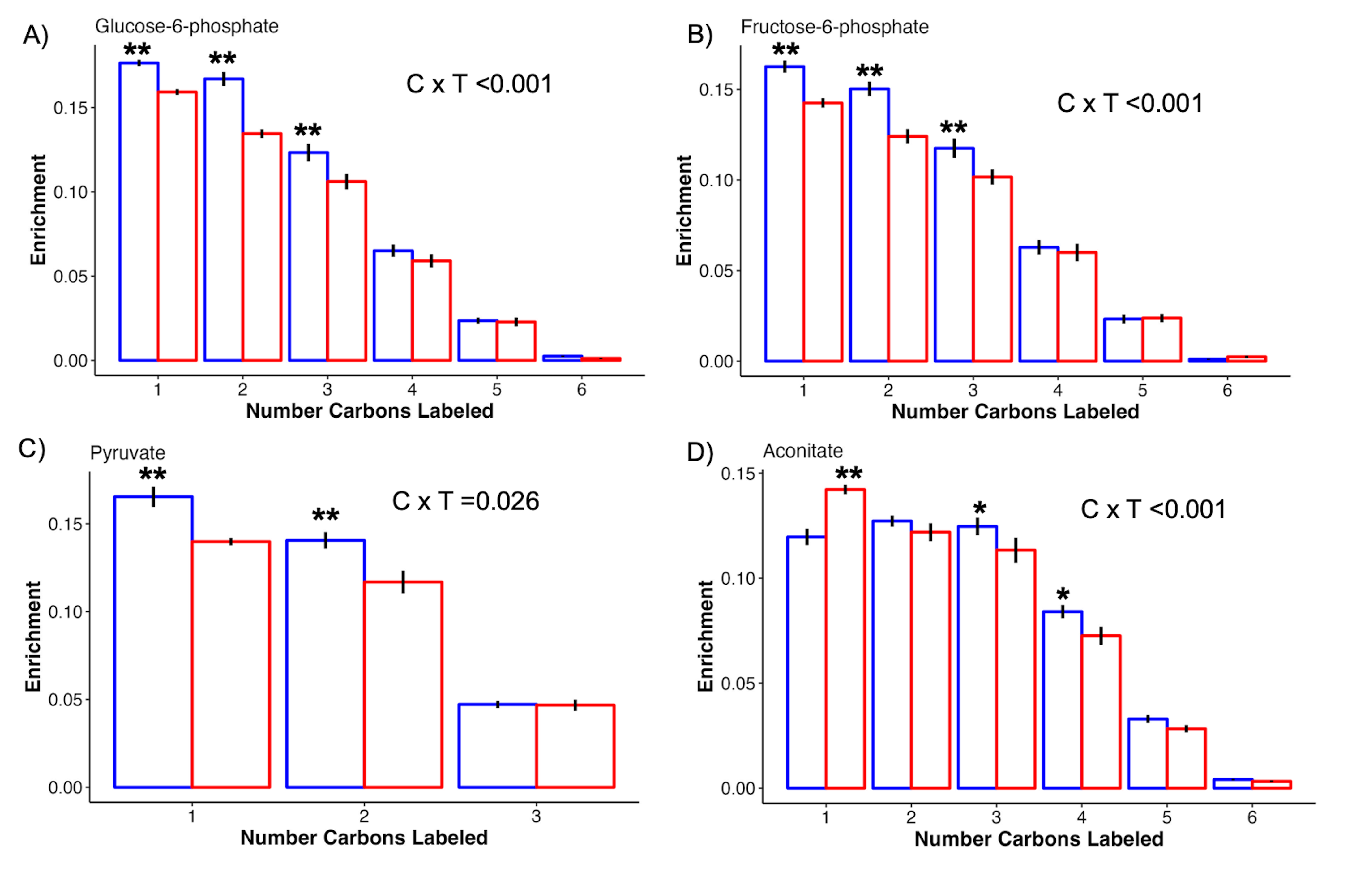

Supplement: S10 Fig — Significance (P-value) of the interaction of number of labeled carbons (“C”) and temperature treatment (“T”) shown in text determined by two-way analysis of variance tests. In all plots, red indicates high temperature and blue indicates ambient temperature. Asterisks indicate significance of post hoc comparisons with * indicating P < 0.05 and ** indicating P < 0.01. No asterisks indicate P > 0.05. The data underlying this figure can be found at 10.5281/zenodo.13835295. (TIFF) [file pbio.3002875.s010.tiff]
