# Supplementary material for: Coral larvae increase nitrogen assimilation to stabilize algal symbiosis and combat bleaching under increased temperature
Source: PLoS Biol. 2024 Nov 12;22(11):e3002875. doi: 10.1371/journal.pbio.3002875 (PMC11556732; doi:10.1371/journal.pbio.3002875)
Supplement: Attachment — Submitted filename: Response_Review.docx [file pbio.3002875.s018.docx]

Thank you for your review and feedback on our manuscript. We are delighted the reviewers feel: “*this manuscript fills an important knowledge gap*”; *“Overall, the manuscript is well written, with an excellent level of detail.”; “Overall, this is a very nice manuscript!”,* and *“Overall, the manuscript is a delight to read and has important data for the field.”*  We have addressed the suggested revisions below.

As requested by the editor, we have changed the title of this manuscript to “Coral larvae increase nitrogen assimilation to stabilize algal symbiosis and combat bleaching under increased temperature”.

Reviewer #1:

The manuscript assesses the role of nutrient cycling dynamics in coral larvae under heat stress. Also, this is a topic that is well studied in adult corals, we are missing baseline data in coral larvae. Search, this manuscript fills an important knowledge gap. The study is very well embedded into the current literature of carbon/nitrogen cycling between coral host and algal symbiont, and how the 'balanced tranfser' is maintaining a stable symbiosis. in this regard, the findings are remarkable: that larvae 'manipulate' the nitrogen availability to keep algal symbionts 'in check'.

What might be useful to add is a discussion on the role of diazotrophs. Admittedly, this study is already vast, so I would not see the necessity to do 16S/diazotroph profiling. However, based on the current literature (e.g., <https://academic.oup.com/ismej/article/16/4/1110/7474332>), change of diazotroph community/activity should be mentioned in the Discussion. This might be particularly interesting in light of a 'maturing' microbiome (winnowing = larvae respond differently than adult counterparts), and the putative high level of species specificity in the heat stress response, as nicely pointed out in the discussion

We greatly appreciate the reviewer’s suggestion to increase our discussion of the role of microbial communities in affecting nutrient metabolism. We have added two statements to the discussion in section B to address this topic and offer suggestions for future work. This section now reads,

“The coral holobiont is a complex nutritional network between host, symbiont, and microbial partners and additional work is required to characterize the role of associated microbial communities in affecting carbon-nitrogen balance in corals. Specifically, additional study of the role of diazotroph microbial communities in affecting nitrogen assimilation in the holobiont is required, especially in light of previous work showing that heat stress increases nitrogen fixation by diazotroph communities (Rädecker et al. 2022). Shifts in the relative role of microbial nitrogen fixation that occur as larvae undergo winnowing of microbial partners throughout development (e.g., Lema et al. 2014) may also contribute to life stage specific metabolic responses to heat stress.”

I was surprised to see that the GitHub repo is not yet publicly available (what harm could be done?), so I trust the authors will make sue everything is not only uploaded to archives but also publicly accessible.

We apologize for any challenges the reviewer experienced in accessing the data. We have ensured that the GitHub repository is indeed publicly available. We have further deposited the data in permanent open repositories with DOI links available at Zenodo and OSF and have issued a new static Release of the GitHub repository. These links are updated in the data availability statement.

It's a rather lengthy read that, however, exercises excellent integration with the available literature (aside from the diazotroph aspect, see above).

------------------------------------------------------------------------

Reviewer #2:

This manuscript describes a detailed metabolomics study of coral larvae under thermal stress. The findings add nicely to those form previous metabolomics studies of corals and other symbiotic cnidarians, both under thermal stress and in response to different Symbiodiniaceae species, by improving our understanding of how the early life stages of corals might respond to, and combat, elevated seawater temperatures. Overall, the manuscript is well written, with an excellent level of detail. The manuscript is quite long (the methods especially so) and, as tends to happen with such large datasets, it takes the reader a little while to grasp all of the complex interactions being described - however, I don't think that this requires any changes from the authors. Overall, I have relatively few comments on the science, as the authors have done an excellent job of explaining and discussing the findings, and I support the manuscript's publication pending a few minor edits:

Thank you for your comments and feedback on our manuscript. We have addressed each recommendation below.

Line 59: Better as "Symbiodiniaceae, which ARE"

Revised as requested.

Line 70: Should be "of lipid reserves..."

Revised as requested.

Lines 89-91: The grammar here is a little odd - what is meant by "time window" (is this even necessary?), while "in" on Line 91 is an error I think.

Revised as requested.

Line 105: Should be "increases...impact metabolism"

Revised as requested.

Line 115: "Falcon" should start with a capital letter.

Revised as requested.

Line 138: Delete "was" to say "treatment that supplied..."

Revised as requested.

Lines 145-149: This part is unclear - I suggest trying to clarify what's meant by the ambient and high temperature treatments - for instance, why are two mean temperatures and sample sizes stated for the ambient treatment in Lines 148-149 - is some information missing here? The same query arises in Lines 241-242.

Thank you for pointing out this inconsistency. We have corrected this statement to now read:

“During the larval thermal exposure, mean temperature (± std. dev.) in the ambient treatment was 26.7 ± 0.4°C (n=1,482) and 29.2 ± 0.6°C (n=1,482; Fig. S1) in the high temperature treatment.”. This has also been corrected in the settlement section as “During the settlement period, mean temperature (± std. dev.) in the ambient treatment was 27.2 ± 0.6°C (n=3,051) and 29.2 ± 0.5°C (n=3,052; Fig. S1) in the high temperature treatment.”

Line 149 (and elsewhere): "PAR" isn't a unit - replace this with "umol photons m-2 s-1".

Revised as requested.

Line 168: Should be "sensor dish readerS"

Revised as requested.

Line 179: End bracket is superscripted

Revised as requested.

Line 192: Delete "were"

We have revised this statement as described below under the recommendation to clarify photosynthesis and respiration rate results. The statement now reads:

“Residual normality and homogeneity of variance were assessed using quantile-quantile plots and Levene’s tests in the car package, respectively [55].” We have therefore retained the word “were” in this revised statement.

Line 217: Do you mean "conical flasks"?

This has been changed to “larval tanks” for clarity.

Line 220: Should be "IS sourced..."

Revised as requested.

Line 250 (and elsewhere in the methods, e.g. Lines 265-266): Should be 600 ul...WERE added..."

This has been corrected throughout. We have also standardized statements of time in the methods section (i.e., “hour” to “h”, “minute” to “min”, and “second” to “sec”).

Line 253: Better as "designated AS the..."

Revised as requested.

Line 254: Better as "for MEASURING symbiont cell..."

Revised as requested.

Lines 299, 303 and 499: "c2" should be italicized.

Revised as requested.

Line 495: How is it possible that Pgross didn't change, R did change, and therefore that there was no change in Pnet given that this parameter is a function of the other two? Was this simply a statistical quirk or is there an error?

Thank you for this feedback. There is not an error in the statistical analysis of this data. P gross is calculated as net oxygen produced (P net) plus oxygen consumed (R). In the previous version of Figure 4, we did not have consistent y-axis limits across all metrics, which did not visualize the difference in each of these metrics well. In this revision, we have changed the y-axis of Fig 4 to be consistent for P gross, P net, and R, which now allows for clearer visualization of how these metrics are calculated.

Line 499: Chlorophyll-a should use an italicized "a", not alpha.

Revised as requested.

Lines 727-728: The grammar is a bit wayward here, so needs to be tidied up.

This statement has been rephrased to:

“Additional metabolic pathways dependent on glycolysis, including generation of lactate through lactate dehydrogenase activity, were also greater in ambient larvae. Generation of lactate may also help to restore oxidized energetic co-factors that are needed for glycolysis [93]”.

Lines 738-739: A similar conclusion about maintained glucose/C translocation during bleaching was reached by Hillyer et al. (2017) New Phytologist. This paper is cited elsewhere, but it's worth noting this particular point here.

We agree and have added the following statement to this section:

“This result is consistent with previous work showing maintained glucose translocation under thermal stress and bleaching in symbiotic *Exaiptasia* (previously *Aiptasia* sp.) anemones using ^13^C metabolomic tracing (Hillyer et al. 2017).”

Line 753: Delete "therefore"

Revised as requested.

Line 767: I don't think it quite right to refer to this as an "aposymbiotic species" or "aposymbiotic coral" - what you mean is a coral with an aposymbiotic stage in its life-history. Perhaps clarify what you mean here.

We have revised this statement to more clearly indicate aposymbiotic life stages by replacing “aposymbiotic species” with “aposymbiotic life stage” and “aposymbiotic early life stages” in this paragraph.

Line 787: "Aiptasia sp" is old taxonomy - replace this with "Exaiptasia diaphana".

Revised as requested.

Line 789: Better as "M. capitata corals downregulate glycolysis".

Revised as requested.

Lines 807-810: There has been a tendency to state this mechanism (N limitation for promoting C translocation) as a fact in the last few years, yet the only study (though I could be wrong!) to directly test this hypothesis (Davy & Cook 2001, Marine Biology) reported no change in %translocation with N limitation. While much more study is still needed on this topic, I suggest some less definitive wording here, and citation of the aforementioned paper, which is currently absent.

We agree with this recommendation. We found that the result of no change in translocation under nutrient limitation induced by starvation an interesting point and have suggested that future work investigate how nutrient limitation alters translocation in feeding life stages (e.g., adults) vs non feeding life stages (e.g., larvae, as in our study). We have changed this statement to now read:

“Nitrogen limitation may favor the translocation of excess photosynthates from the symbiont mediated by changes in symbiont density and population growth [4,7,22,26,28]. However, when nitrogen availability increases and symbiont population growth increases, symbionts may retain more photosynthates for themselves, decreasing translocation to the host [7,9,27,29]. In contrast, previous work in symbiotic *Exaiptasia* anemones found no change in carbon translocation under nutrient limitation induced by starvation (Davy and Cook 2001), demonstrating the need for additional work to clarify how nutrient limitation affects translocation in cases of starvation during feeding life stages as compared to non-feeding larval stages.”

Discussion section B: A recent study that may or may not be relevant to this part of the discussion is Crehan et al. 2024, J. Exp. Biol., that describes the impacts of thermal stress on the assimilation of inorganic and organic N sources.

Thank you for recommending this publication. We found that the results are particularly interesting in the context of shifts in the urea cycle seen in our results. We have therefore added the following statement to the last paragraph of section B in the discussion:

“Under heat stress conditions, *S. pistillata* corals exhibited increased host assimilation of urea even though assimilation of ammonium was disrupted in both the symbiont and host (Crehan et al. 2024), further demonstrating that relative usage of urea and ammonium metabolism can shift under bleaching conditions”.

Line 837: Should it say "THOSE of" rather than "that of"?

Corrected by changing to “were more enriched than..”

Line 862: Better as "THE larval" rather than "our larval".

Revised as requested.

Overall, this is a very nice manuscript!

------------------------------------------------------------------------

Reviewer #3:

The authors attempt to determine how heat impacts coral host metabolism during heat stress. Overall, the manuscript is a delight to read and has important data for the field. The writing is done well and handles a complex metabolic experiment with ease. Specifically, the authors examine metabolite levels and 13C incorporation in symbiotic M. capitata larvae after 3.5 days of either ambient or high temperature. They chose temperatures that are 'ecologically relevant'; however, I will note that this is not needed for good laboratory experiments. The high-temperature treatment had no impact on survival, symbiont density, or algal photosynthesis, suggesting that the animals are not bleaching during this window. The authors find that the heat-exposed larvae have an overall decrease in metabolism. This conclusion is supported by lowered respiration of the host and decreased 13C enrichment in glycolysis and TCA cycle intermediates. Even though metabolism is disrupted, carbon

translocation from the algae to the host is sustained. Additionally, this translocated carbon is found enriched in glutamine and glutamate because of increased ammonium assimilation. Overall, the authors conclude that the host reserves/increases its glucose levels under these heat conditions and maintains its nutritional symbiosis with the algae by increasing ammonium assimilation and nitrogen sequestration.

Thank you for your feedback and review of our manuscript. We have addressed the request for revisions as detailed below.

Major comments:

The manuscript is overall good. However, I recommend some minor changes that may be helpful to the reader.

First, the paper's title suggests there is data showing that nitrogen sequestration mechanisms are essential to stabilizing carbon provisioning from algal symbionts under increased temperature. I believe this was more of a hypothesis generated in the discussion. The title, as is, oversteps the data.

Thank you for this feedback. We have adjusted the title as requested by the editor to now read “Coral larvae increase nitrogen assimilation to stabilize algal symbiosis and combat bleaching under increased temperature”

Second, in the abstract, Lines 37-39 also overstep the data. There are no functional data supporting this claim. This conclusion appears to be a hypothesis from the data.

We agree that this is a hypothesis statement and it has been revised to state:

“Host nitrogen assimilation via dipeptide synthesis appears to be used for nitrogen limitation to the Symbiodiniaceae, and we hypothesize that nitrogen limitation contributes to the observed reduction in symbiont population growth and retention of fixed carbon by favoring photosynthate translocation to the host.”

Also, in Lines 40-42, this conclusion is based on the correlation between the metabolic trends and the lack of bleaching. These changes may or may not impact bleaching. Minor changes to the abstract and discussion would help highlight some degree of uncertainty.

We agree with the reviewer. We have altered this statement to now read:

“Collectively, our findings indicate that although these larvae are susceptible to metabolic stress under high temperature, diverting energy to nitrogen assimilation to maintain symbiont population density, photosynthesis, and carbon translocation may allow larvae to avoid bleaching”.

Third, a key finding in the paper, Discussion Lines 873-888, seems to conflict with refs 21 and 22. The difference could be due to different coral life stages, species, or other reasons. As these results challenge major correlative conclusions made by the other references, these conflicting results should be a key point of the paper and the abstract.

Thank you for this feedback. We have revised this section of the discussion to clarify points of contrast and offer additional explanations for this contrast. The section now reads:

“Our study advances the generality of this carbon-nitrogen feedback loop framework across life stages (Wang and Douglas 1998; Cui et al. 2019; Cui et al. 2023; Rädecker and Meibom 2023; Rädecker et al. 2023), by clearly demonstrating in larvae that symbiont derived carbon is incorporated into amino acids involved in ammonium assimilation. However, there are deviations from the mechanisms described in adult corals. Specifically, we found that the larval coral host increased ammonium assimilation without a concurrent increase in central carbon metabolism, in contrast to previous findings that elevated ammonium assimilation occurs alongside increased carbon metabolism (Cui et al. 2023; Rädecker et al. 2023). Rather, we observed lower central carbon metabolism paired with higher ammonium assimilation. An alternative explanation of our observation of lower central carbon metabolism paired with higher ammonium assimilation in contrast to previous work could be due to lag effects in metabolic response to symbiont growth and translocation. For example, it is possible that in the early stages of temperature exposure, carbon translocation is reduced, resulting in lower rates of carbon metabolism through glycolysis (Fig. 8-2). In response to decreased translocation, energy savings could then be diverted to ammonium assimilation and dipeptide synthesis (Fig. 8-3), leading to reduced symbiont population growth and stimulation of carbon translocation back to normal levels (Fig. 8-1). Following return to homeostasis, the host would then be able to restore normal carbon metabolism rates, but it is possible that we did not yet observe this change in the temporal scale of our sampling. Determination of the temporal unfolding of this mechanism would require a high frequency time series from the initial to the bleaching states. Further, the contrast between our results and previous may be due to differences in metabolic responses to stress in larvae as compared to adult corals and requires additional work exploring the mechanisms of carbon-nitrogen feedback loops across life stages.”

We have also added the following statement to the abstract to highlight potential life stage specific responses:

“Collectively, our findings indicate that although these larvae are susceptible to metabolic stress under high temperature, they can combat bleaching by diverting energy to nitrogen assimilation in order to maintain symbiont population density, photosynthesis, and carbon translocation may allow larvae to avoid bleaching and highlight potential life stage specific metabolic responses to stress.”

Minor comments:

In the introduction, the breakdown of nutrient exchange is treated as a known mechanism driving bleaching. However, this has not been formally tested and remains a model. There are other models as well. It is worth clarifying that it is a model (e.g., lines 58-63).

We have revised this section to now read:

“Coral bleaching under high temperature is characterized by the loss of the Symbiodiniaceae, which are the coral’s primary source of nutrition [13–15], and can result in mass mortality [11,16–18]. The mechanisms underlying the onset and progression of coral bleaching are complex and a topic of intense scientific study (see a recent review in [19]). Research in the last two decades has demonstrated that oxidative stress [19,20] in addition to carbon and nitrogen imbalance models may explain dysbiosis that occurs under bleaching conditions [19,21–25].”

Similarly, conclusions in Lines 64-75 could be made stronger by highlighting which are strongly supported by the data vs. hypothesized mechanisms. The data in the paper fill in some of these holes. However, it is unclear where the outstanding questions are from reading the introduction.

Thank you for this recommendation. We agree that the manuscript introduction requires stronger statements on current knowledge gaps. We have revised this paragraph to now include the following text:

“Conceptual models propose that the decoupling of carbon and nitrogen cycling can lead to breakdown of the symbiotic relationship under thermal stress (Rädecker et al. 2021). Recent experimental evidence from adult Exaiptasia anemones and reef building corals demonstrate that the supply of carbon regulates nitrogen availability to the symbiont, supporting the role of coupled carbon-nitrogen metabolism in generating a stable symbiotic state under ambient conditions (Rädecker et al. 2023; Cui et al. 2023). There is a critical need for experimental research to test conceptual models and investigate the role of nutrient cycling on the breakdown of the symbiotic relationship under elevated temperatures. Further, key knowledge gaps remain on how these processes vary across life stages, especially in early life stages during which the symbiotic relationship is established. Therefore, in this study we use stable isotope metabolomic tracing to investigate carbon and nitrogen metabolic feedbacks and shifts in symbiotic nutritional exchange under thermal stress in a symbiotic early life stage.”

In Figure 1, there is no indication of the isotope treatment along the timeline of the experiment.

In figure 1, sampling of metabolomics occurred at the dotted line labeled as “sampling”. We have adjusted the figure caption to now include the statement:

“Sampling of larvae occurred at ~7 dpf (165 hpf) for metabolic rates, physiological assays, and stable isotope metabolomics (dotted line).”

Line 496: What do the authors mean by "trend for an elevated P:R ratio".

This statement has been clarified to now read:

“This resulted in a trend for an elevated P:R ratio in exposed larvae due to reduced respiratory rates, but this was not statistically significant (Fig. 4D; P=0.054).”

Line 504: Has a test for normality been done on S3D? It looks like the data might be bimodal, suggesting a different statistical test be used.

Host carbohydrate content data (Fig S3D) were assessed using qq plots and homogeneity was assessed using Levene’s tests. These data satisfied the assumptions and were therefore analyzed using a Welch t-test.

To clarify the methods used for analysis, we have added a statement of how each physiological metric was analyzed in the “physiology data analysis” section of the methods. This section now reads:

“For all physiological analyses, the effect of treatment on each metric was tested with two-sample Welch t-tests in R. Residual normality and homogeneity of variance were assessed using quantile-quantile plots and Levene’s tests in the car package, respectively [55]. Holobiont carbohydrate content per Symbiodiniaceae cell (ug cell^-1^) was the only metric analyzed using a non-parametric Wilcoxon rank sum exact test due to violation of homogeneity of variance.”

Line 517: No reference to the data.

We have now included the full data for this statement in Table S2 and have cited this table in the text.

Line 450: Should there be a citation for the multivariate PERMANOVA tool?

We have now cited the *vegan* package in R in this sentence.

Line 519 States a significant difference in the multivariate metabolite pool sizes between ambient and high-temperature larvae for both 12C and 13C. However, when referencing the S5A figure, there is no comparison of temperature. I believe they meant to refer to S4A.

Thank you for identifying this error. We have corrected the figure citation to Figure S4.

Line 522: No reference to supplemental data.

We have now included the full data for this statement in Table S2 and have cited this table in the text.

Line 605: no reference to the data.

We have now referenced Table S3 in this statement.

Line 607-609: inconsistent referencing to data.

Thank you for pointing out this inconsistency. We have added reference to supplementary tables for this statement. Throughout the results, we cite VIP values for non-significant metabolites to illustrate the metabolites that did not change as a result of treatment. We did not previously cite a supplementary table of these values and have now added two supplementary tables to supply this data (Table S5 for pool size VIP values; Table S7 for enrichment VIP values <1). These supplementary tables have been referenced throughout the results and other supplementary tables have been renumbered as required after this revision.

Line 670: citation for maintaining the symbiont population in a nitrogen-limited state.

We have added the relevant references to this statement.

Line 842: "… in the high [] treatment.."

We have added the word “temperature” to now read “high temperature treatment”.

Line 842: no reference to figure for the upregulation of the urea cycle, particularly the mitochondria.

We have now cited Fig. 7D in this statement.

Line 871: "…growth rate declines this would allow for…" needs to be rewritten.

We have corrected this statement to now read:

“...consequently, declines in symbiont population growth rate could favor greater translocation of excess carbon…”
